# 

**Published:** 1887-11

**Authors:** 


					﻿NOVEMBER, 188*7-
OLD SERIES
VoU xxv
NEW SERI0
Vol. IV.—No. 9,*
1855
sfe
lEDIGAl^M
*•Vpw.f.westmorel'anonBJ
H.VlVLMlLLER.M.D.LL.D.j^
Op*	JAMES A.GRAY, M.D.^|
Crushed By ja^rh^ison&c^
fc	Atlanta .GA.
8UB8CRIPTION: S2.8O A YEAR.
				

